# Tumor suppressor DEAR1 regulates mammary epithelial cell fate and predicts early onset and metastasis in triple negative breast cancer

**DOI:** 10.1038/s41598-022-22417-4

**Published:** 2022-11-14

**Authors:** Uyen Q. Le, Nanyue Chen, Seetharaman Balasenthil, Eugene Lurie, Fei Yang, Suyu Liu, Laura Rubin, Luisa Maren Solis Soto, Maria Gabriela Raso, Harsh Batra, Aysegul A. Sahin, Ignacio I. Wistuba, Ann McNeill Killary

**Affiliations:** 1 Department of Translational Molecular Pathology, The University of Texas M.D. Anderson Cancer Center, Houston, TX 77030 USA; 2 Department of Biostatistics, The University of Texas M.D. Anderson Cancer Center, Houston, TX 77030 USA; 3 Department of Pathology, The University of Texas M.D. Anderson Cancer Center, Houston, TX 77030 USA

**Keywords:** Breast cancer, Cancer genetics, Cancer stem cells, Tumour-suppressor proteins, Cancer

## Abstract

Triple negative breast cancer (TNBC) is a disease of poor prognosis, with the majority classified as the basal-like subtype associated with epithelial-mesenchymal transition and metastasis. Because basal breast cancers originate from proliferative luminal progenitor-like cells upon dysregulation of proper luminal differentiation, genes regulating luminal-basal transition are critical to elucidate novel therapeutic targets to improve TNBC outcomes. Herein we demonstrate that the tumor suppressor DEAR1/TRIM62 is a critical regulator of luminal cell fate. DEAR1 loss in human mammary epithelial cells results in significantly enhanced mammosphere formation that is accelerated in the presence of TGF-β/SMAD3 signaling. Mammospheres formed following DEAR1 loss are enriched for ALDH1A1 and CK5 expression, EpCAM^−^/CD49f^+^ and CD44^high^/24^low^ basal-like epithelial cells, indicating that DEAR1 regulates stem/progenitor cell properties and luminal-basal progenitor transition. We show that DEAR1 maintains luminal differentiation as a novel ubiquitin ligase for SNAI2/SLUG, a master regulator driving stemness and generation of basal-like progenitor populations. We also identify a significant inverse correlation between DEAR1 and SNAI2 expression in a 103 TNBC case cohort and show that low DEAR1 expression significantly correlates with young age of onset and shorter time to metastasis, suggesting DEAR1 could serve as a biomarker to stratify early onset TNBCs for targeted stem cell therapies.

## Introduction

Triple negative breast cancer (TNBC) is a highly aggressive form of breast cancer lacking expression of the estrogen and progesterone receptors as well as the master oncogene HER2 (ER^−^, PR^−^, and HER2^−^). TNBC has a high risk of metastasis, a poor prognosis and lacks efficacy for conventional targeted therapies commonly utilized for treatment of ER^+^/PR^+^ and HER2^+^ breast cancer subtypes^1^. Once TNBC progresses to metastasis, the median survival is only 13 months; ultimately, metastatic TNBC patients die from their disease. TNBC also tends to occur in patients of younger age, making it a high priority for the field to identify predictive biomarkers to stratify TNBCs for targeted therapies as well as early detection strategies to prevent metastasis from this devastating disease^2,3^.

A contributing factor to the dismal prognosis associated with TNBC is its intratumor heterogeneity, derived from both differentiation-state heterogeneity and cellular-state plasticity, which hinders the effectiveness of therapeutic treatments^4^. The normal adult mammary gland epithelium controls the differentiation and organization of a variety of epithelial cell states therein such as basal, mature luminal, and luminal progenitor cell populations. While the exact differentiation hierarchy of these cell types is still unclear, studies suggest that they share common progenitors and can transition from one to another^5^. Genetic and microenvironmental perturbations alter the normal mammary epithelial differentiation cascade, leading to changes in cellular reprogramming, cellular plasticity, transitions, and shifts in occupancies of various cellular states within the differentiation landscape^6–10^. As a result, a single tumor contains mixtures of phenotypically diverse cell subpopulations that transition along various positions in a multidimensional scale along luminal to basal, differentiated to stem-like, and epithelial to mesenchymal axes^11,12^. Where a cell falls within this space, the dynamics of which are shaped by the particular genetic or microenvironmental influences, is thought to determine its distinct role (i.e. in proliferation, invasion, etc.) in tumorigenesis^13^, as well as its sensitivity to therapies^14^. The co-occurrence of cells across various cell states within a given tumor population has been observed in breast cancer, made possible by the resolution afforded by in vivo mouse models and single cell experiments, suggesting a much more plastic tumor cell state occupying partial or intermediate/hybrid epithelial-mesenchymal transition (EMT) states along the EMT spectrum and could better explain the interplay between stemness and EMT^15,16^.

The vast majority of TNBCs are categorized as overlapping with the basal-like breast cancer (BLBC) subtype, which is characterized by the expression of basal/myoepithelial signatures and mesenchymal markers suggestive of EMT occurrence^10,17–19^. The BLBC subtype is thought to originate from proliferative luminal progenitor-like cells, which harbor driver mutations (e.g. *BRCA1*) causing their dedifferentiation and transition towards a basal state^10,20^. Luminal progenitor-like epithelial subpopulations in normal breast cells can spontaneously generate mesenchymal-like cells with partial myoepithelial traits, and invasive behavior, through EMT and inhibition of luminal differentiation by SNAI2 and ZEB1^21^. The inherent phenotypic plasticity in normal progenitor cells, maintained through the inhibition of luminal cell differentiation by factors such as SNAI2^6^, is exploited in a cancerous state for the generation of a functionally diverse set of cell states required for the various stages of tumorigenesis (e.g. invasion and metastasis)^6,21^.

TRIM family member proteins have been shown to play a role in regulating acquisition and maintenance of stem-like states, including in cancer cells, through their regulation of core transcription factors^22–25^. We previously identified the E3 ubiquitin ligase DEAR1/TRIM62 as a tumor suppressor and master regulator of acinar morphogenesis and cell polarity, as well as a negative regulator of TGF-β-mediated EMT and migration in immortalized, non-tumorigenic HMECs^26,27^. DEAR1 negatively regulates the expression of genes related to EMT and mammary epithelial cell fate, such as *SNAI1/2*, *ZEB1/2*, and *TWIST*, via ubiquitination of their upstream activator SMAD3^26,28,29^. Interestingly, DEAR1 expression by immunohistochemistry in an early onset, node negative breast cancer cohort (n = 123) correlated with 95% local recurrence free survival over 15 years post-surgery as well as loss of DEAR1 expression significantly correlated with the TNBC phenotype (52/123 cases)^27^. Altogether, our previous work led us to hypothesize that DEAR1 loss is associated with improper luminal cell fate specification.

In the present study, cumulative results document compelling evidence that DEAR1 is an important, novel regulator of luminal cell fate and also provide evidence for its potential clinical utility as a biomarker to stratify TNBC patients for risk of metastasis or targeted therapies aimed at the pathways and cell plasticity regulated by DEAR1.

## Results

### Loss of DEAR1 expression in HMECs enhances primary mammosphere formation

**Figure 1:**
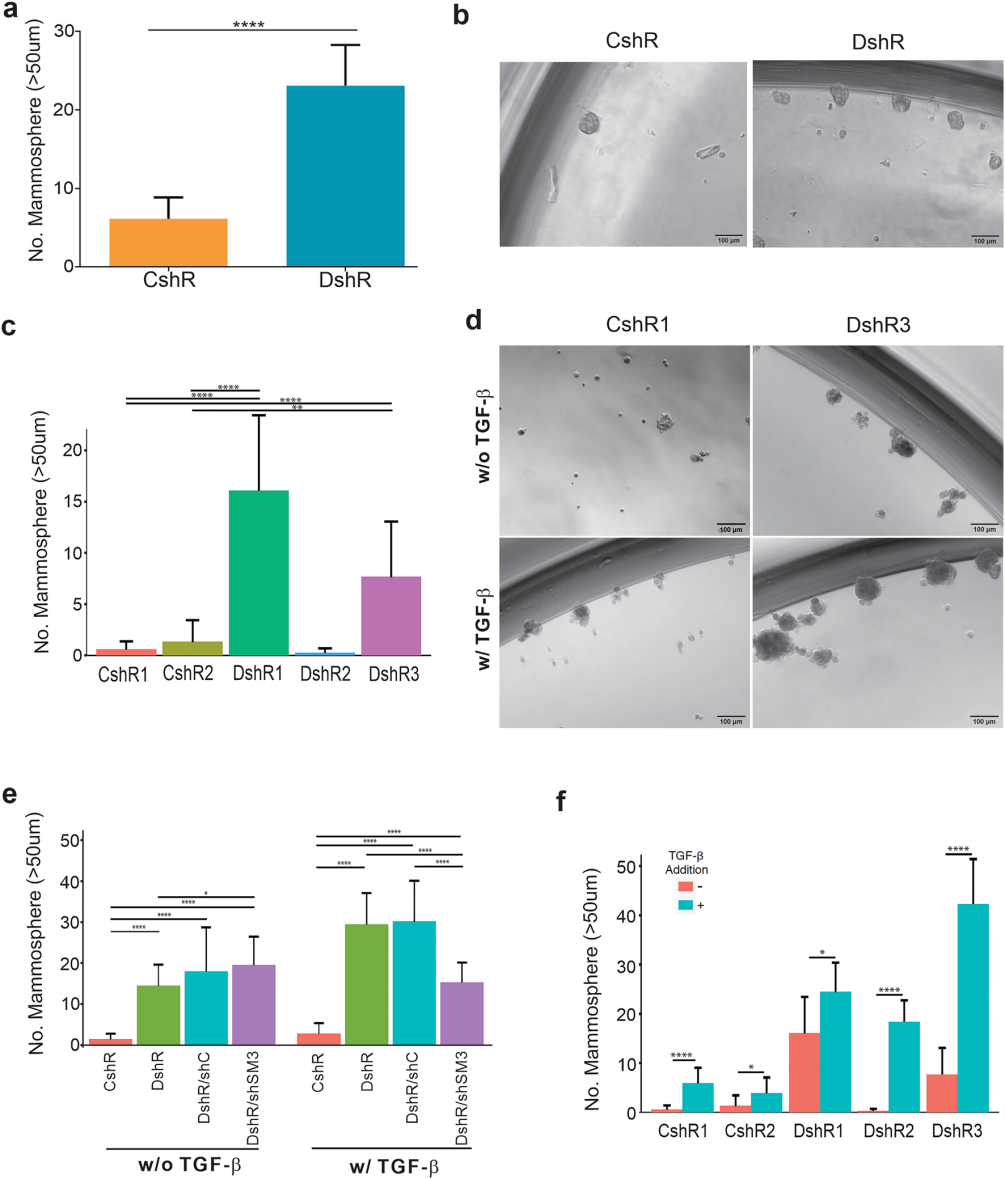
Stable *DEAR1*-KD in HMECs results in increased numbers of mammosphere independent of SMAD3 expression. (**a**) Representative bar plots showing mean ± SD counts of primary mammospheres (> 50um) from control vector (CshR) and *DEAR1*-KD (DshR) MCF10A cells across three trials. Each cell line was grown in at least 8 wells of 96-well plate in mammosphere growing conditions for 17 days for each individual trial. P-values were calculated using a two-tailed Student t-test between groups. **** p < 0.0001. (**b**) Representative images of mammospheres from (**a**) taken using a Zeiss microscope at ×10 magnification (Scale bars equal 100 µm). (**c**) Bar graphs representing the mean ± SD number of mammospheres (> 50 µm) formed from two control vector clones (CshR) and three *DEAR1-*KD clones (DshR) from 76N-E6 cells grown in mammosphere-growing conditions for 14 days across three trials. P-values were calculated using Wilcoxon Rank Sum tests. **p < 0.01 and ****p < 0.0001. (**d**) Representative images at ×10 magnification of CshR1 (left) and DshR3 (right) mammospheres quantified in panel (**c**) in the absence (top) and presence (bottom) of TGF-β (Scale bars equal 100 µm). (**e**) Bar plots showing the mean ± SD number of mammospheres formed from CshR, DshR, DshR/shC, and DshR/shSM3 cells without (left) and with (right) TGF-β treatment across 3 trials. p-values calculated using Wilcoxon Rank Sum tests. *p < 0.05–0.1 and ****p < 0.0001. (**f**) Bar graphs represent the mean ± SD number of mammospheres (> 50 µm) formed from two control vector clones (CshR) and three *DEAR1-*KD clones (DshR) from 76N-E6 cells treated with TGF-β and grown in mammosphere growing conditions for 14 days across three trials. P-values were calculated using Wilcoxon Rank Sum tests. *p < 0.05–0.1 and ****p < 0.0001. The barplots in (**c**), (**e**) and (**f**) were created using RStudio (Version 1.0.143), https://www.rstudio.com.

Previous studies have provided strong evidence that inhibition of luminal differentiation by *SNAI2* and *ZEB1* leads to maintenance of luminal-progenitor-like states in HMECs^21^. Because our previous studies suggested that loss of DEAR1 resulted in increased SMAD3 and concomitant upregulation of master EMT transcriptional activators^26^, we hypothesized that the tumor suppressor DEAR1 plays a role in maintaining the luminal differentiation state in HMECs. To address this hypothesis, we utilized the mammosphere assay to examine DEAR1’s role in regulating populations with stem or progenitor potential. Stable lentivirus-mediated *DEAR1* knockdown (KD) clones in MCF10A (MCF10A-DshR)^26^ were propagated under mammosphere culture conditions with each cell line grown in at least 8 wells of 96-well plates for 17 days for each individual trial. Results (Fig. 1a,b) document mean counts from three separate trials/cell lines and indicate that controls with wild type DEAR1 expression formed few mammospheres; however, a significant increase in mammosphere formation was observed in MCF10A-DshR (average of 3.8 fold increase) compared to control vector cells (MCF10A-CshR) (23.11 ± 5.18 compared to 6.13 ± 2.75, p-value < 0.0001) (Fig. 1a,b). We conducted a similar experiment using 3 *DEAR1* KD 76N-E6 human mammary epithelial cell (HMEC) (E6-DshR) clones^26^ in which 2 of 3 *DEAR1* KD clones (DshR 1 and DshR 3) generated a significantly higher number of mammospheres compared to both control vector clones (p < 0.05, Fig. 1c,d). Results indicate that DEAR1 expressing E6-CshR HMECs maintain the luminal differentiation state; however, loss of DEAR1 expression drives significant stem/progenitor cell activity in two different HMEC cell lines.

We have previously established that DEAR1 blocks TGF-β-induced EMT by binding specifically to SMAD3, resulting in its polyubiquitination, and that loss of DEAR1 unbridles the TGF-β-SMAD3 axis to elicit SMAD3-directed EMT^26^. To determine if the mammosphere formation is dependent on SMAD3 signaling, we utilized *DEAR1-SMAD3* double KD HMECs (E6-DshR/shSM3) that we previously reported^26^ to perform mammosphere assays. We then analyzed the mean number of mammospheres formed from CshR, DshR, DshR/shC, and DshR/shSM3 cells without TGF-β treatment across three trials (Fig. 1e left panel). Results indicated that loss of DEAR1 (DshR) significantly enhanced mammosphere formation, but in double knockdowns, loss of SMAD3 did not rescue mammosphere formation to control levels (p < 0.0001, Fig. 1e left panel). There was no significant difference in the number of mammospheres formed from E6-DshR/shSM3 cells compared to *DEAR1* KD *SMAD3* shRNA controls (E6-DshR/shC) (19.46 ± 6.95 vs. 17.92 ± 10.75, p-value = 0.345, Fig. 1e left panel). Thus, downregulation of the DEAR1/SMAD3 axis did not fully rescue mammosphere formation, indicating that DEAR1 functions in part independently of the TGF-β-SMAD3 axis to negatively regulate stem or progenitor functions in HMECs.

### TGF-β accelerates the stem/progenitor phenotype in DEAR1 KD HMECs

Because published evidence supports a connection between EMT-inducers and progenitor states^21^, we examined the effect of TGF-β on mammosphere number in the same experiment using *DEAR1* KD E6-DshR HMECs and controls with and without prior culture with TGF-β to determine if loss of DEAR1 in the presence of TGF-β would further increase mammosphere formation. Results indicated that treatment of 76N-E6 *DEAR1* wild type control cells with 4 ng/mL of TGF-β for 48 h prior to plating significantly increased mammosphere numbers compared to control HMECs without TGF-β treatment, although the total number of mammospheres formed remained low (p < 0.0001 and p < 0.05, Fig. 1f). Results demonstrate that DEAR1 maintains HMEC luminal state in control clones in which most cells failed to form mammospheres, although an increase in number was observed following supplementation of cultures with TGF-β. *DEAR1* KD in the presence of TGF-β resulted in significantly increased mammosphere numbers compared to *DEAR1* KD cells without exposure to TGF-β in 3/3 clones (p < 0.05, p < 0.0001, and p < 0.0001, respectively Fig. 1f), indicating that loss of DEAR1 enhances the stem cell/progenitor phenotype which is further amplified in the presence of TGF-β.

To determine whether the increase in mammosphere number in response to TGF-β was directed through SMAD3, mammosphere growth in TGF-β-treated *DEAR1-SMAD3* double knockdown cells (E6-DshR/shSM3) and *DEAR1* KD *SMAD3* shRNA controls (E6-DshR/shC) was also analyzed. In the presence of TGF-β, E6-DshR/shSM3 cells formed significantly fewer primary mammospheres compared to E6-DshR/shC vector controls (15.25 ± 4.91 v. 30.13 ± 10.01, p < 0.0001, Fig. 1e right panel); however, mammosphere formation was not completely rescued to HMEC control levels (p < 0.0001, Fig. 1e, right panel). This partial rescue of mammosphere formation in double knockdown cells provides evidence that, when DEAR1 expression is lost, the TGF-β-SMAD3 pathway contributes to the stem/progenitor phenotype, but it is not the sole pathway leading to a stem/progenitor cell state. Thus, DEAR1 plays a role in regulating luminal differentiation both through its regulation of the TGF-β pathway and also independently of this axis.

### Loss of DEAR1 generates mammospheres enriched for progenitor cell activity

**Figure 2:**
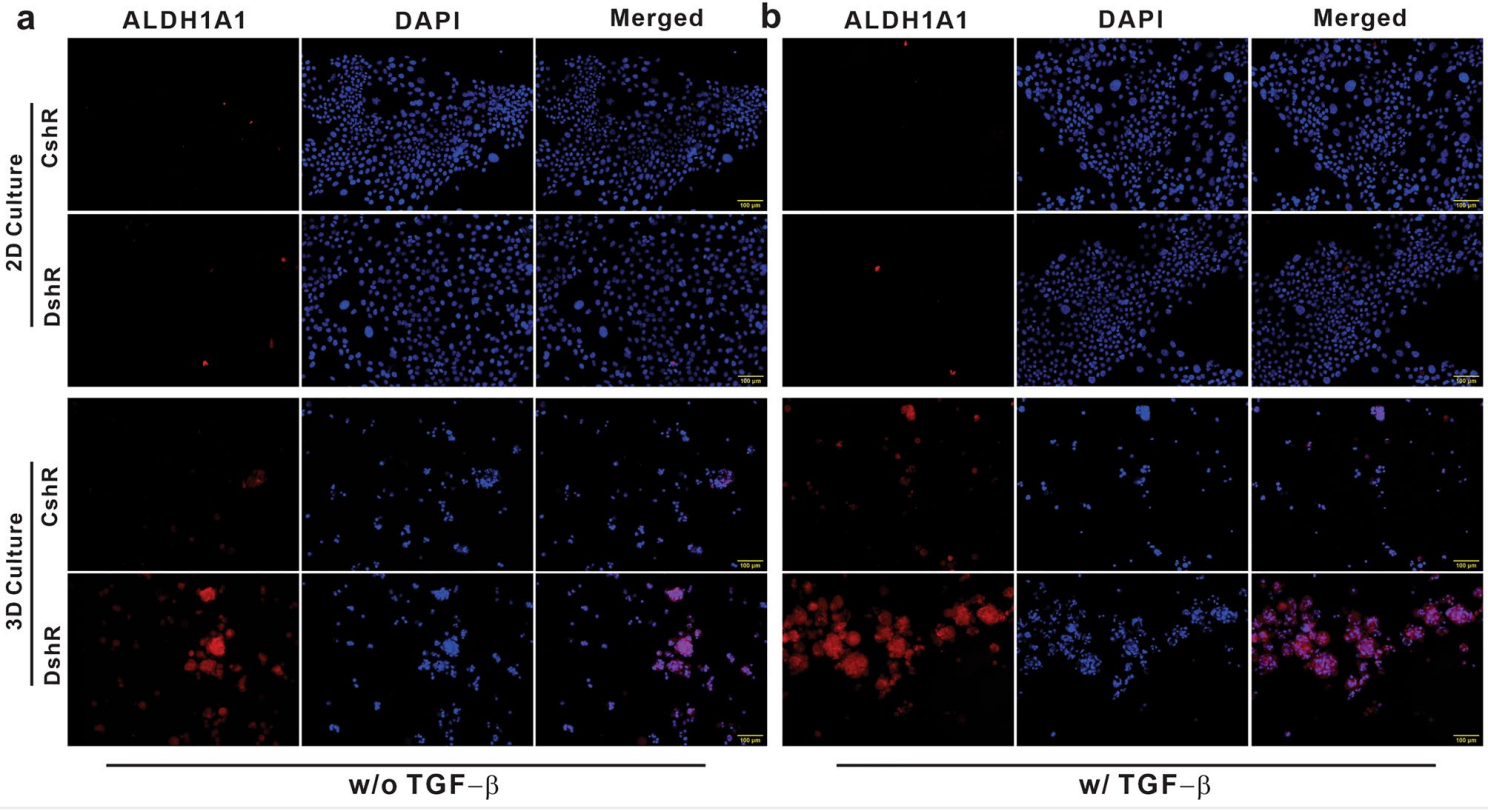
The effect of *DEAR1*-KD on ALDH1A1 expression in 2D culture and 3D mammosphere conditions. (**a**) ALDH1A1 (red) and DAPI (blue) staining of 76N-E6 control (CshR) and *DEAR1-*KD clones (DshR) grown in 2D (top) or 3D (bottom) mammosphere culture in the absence of TGF-β. (**b**) ALDH1A1 staining of CshR and DshR clones grown in 2D (top) or 3D (bottom) mammosphere culture in the presence of TGF-β. Fluorescence images were taken using OLYMPUS cellSens Software. Scale bars equal 100 µm.

Increased mammosphere formation results from stem/progenitor cell activity and the expansion of cells with mesenchymal/myoepithelial features^30^. To determine if DEAR1 plays a role in the regulation of stem/progenitor activity, we examined *DEAR1* E6-CshR and E6-DshR HMEC clones for expression of aldehyde dehydrogenase (ALDH1A1), an established marker of stem/progenitor cells that is overexpressed in both normal and cancer stem cells^31^. Results indicated that, in the absence of TGF-β, ALDH1A1 expression was barely detectable in both E6-CshR and E6-DshR clones grown in 2D culture conditions (Fig. 2a, in 2D culture panels). However, E6-DshR *DEAR1* KD clones in 3D mammosphere conditions showed a clear increase in ALDH1A1 signal (Fig. 2a in 3D culture panels). Furthermore, TGF-β treatment resulted in further elevation of ALDH1A1 expression in 3D culture (Fig. 2b, right panel). These data indicate that DEAR1 deficiency plays a more important role in inducing stem/progenitor properties in the more intrinsic 3D culture conditions rather than in 2D conditions in TGF-β-independent and dependent ways. Interestingly, a small fraction of control HMECs E6-CshR also stained positive for ALDH1A1 in both 2D and 3D culture (Fig. 2a,b), as has been reported^21^.

To determine if loss of DEAR1 contributes to self-renewal, primary mammospheres were passaged through dissemination of spheres into single cell suspension and re-plated at the same density to form secondary and tertiary mammospheres. Mammosphere forming efficiency (MFE) was calculated as a percentage of the number of mammospheres larger than 50 µM/total number of single cells seeded per well. Results indicated that in the absence or presence of TGF-β, loss of DEAR1 expression in HMECs did not result in an increase of the MFE with increasing passages from primary mammospheres (Supplementary Table S1). Thus, our data indicates that loss of DEAR1 in HMECs does not result in stem cell self-renewal, a major stem cell phenotype; rather, DEAR1 plays a role in regulating progenitor cell properties or state occupancy as *DEAR1*-KD resulted in many more mammospheres expressing ALDH1A1.

### Loss of DEAR1 expands subpopulations of cells expressing basal markers

**Figure 3:**
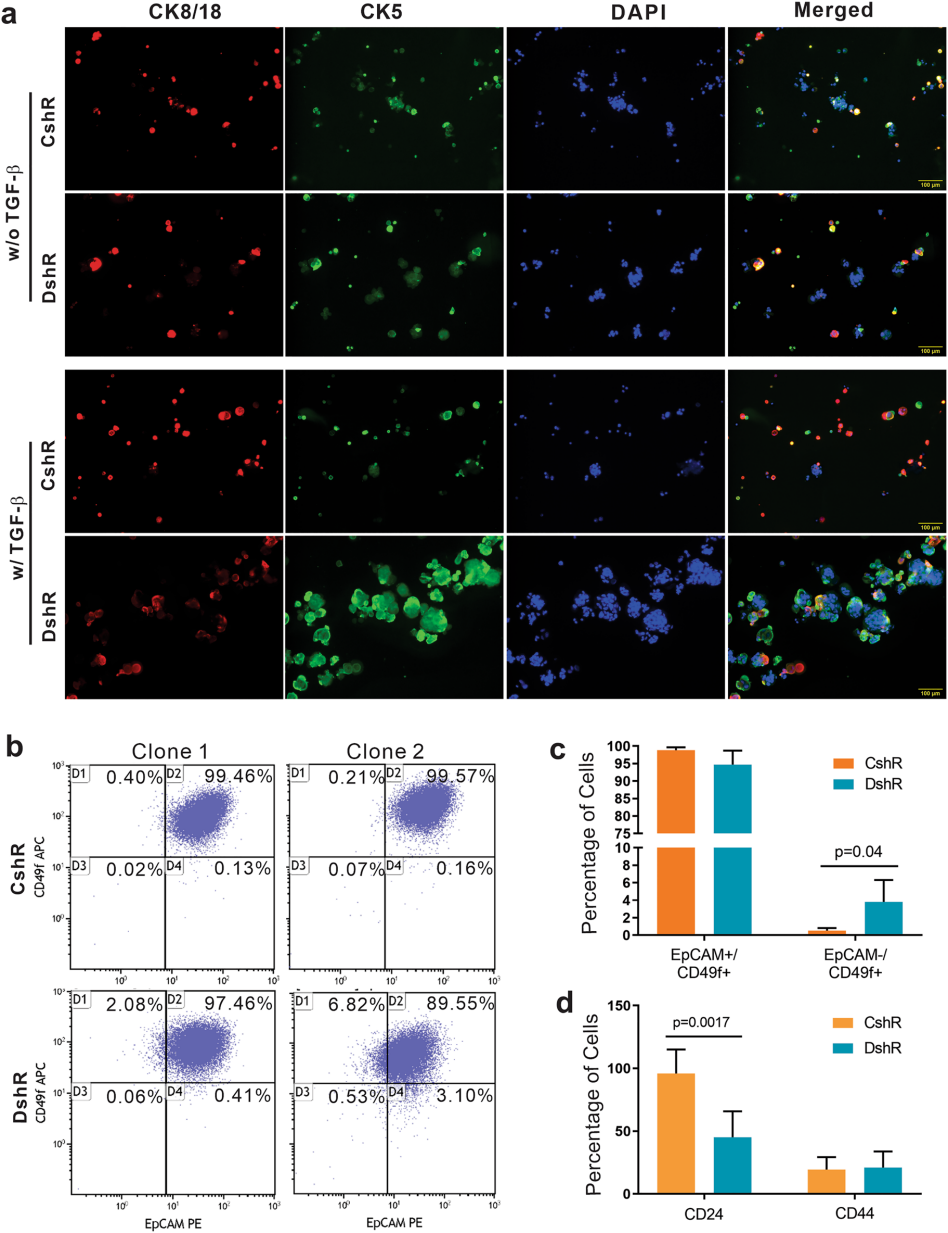
The effect of *DEAR1*-KD on luminal and myoepithelial/basal surface marker expression in HMEC cells. (**a**) Images at ×10 magnification of staining of 76N-E6 control (CshR) and *DEAR1*-KD (DshR) clones grown in 3D mammosphere culture in the absence (top) or presence (bottom) of TGF-β with luminal and myoepithelial/basal cytokeratin surface markers, CK 8/18 (red) and CK 5 (green), respectively. Fluorescence images were taken using OLYMPUS cellSens Software and scale bars equal 100 µm. (**b**) FACS data showing proportion of cells that express EpCAM and CD49f from 2 CshR clones (top) and 2 DshR clones (bottom). (**c**) Bar plots showing the mean ± SD percentage of cells displaying luminal (EpCAM^+^/CD49f^+^) and basal (EpCAM^−^/CD49f^+^) surface markers from CshR (orange) and DshR (teal) clones. p-values were calculated using a two-tailed Student t-test between groups. (**d**) Bar plots quantifying FACS data assaying CD24 and CD44 protein expression from CshR (orange) and DshR (teal) clones. p-values were calculated using a two-tailed Student t-test between groups.

Epithelial progenitor populations in human breast tissue primarily consist of luminal, basal and bipotent progenitor cells^32^. Because epithelial ALDH-expressing proliferative cells are known to display multi-lineage differentiation capacity^13^, we next assayed the expression of markers of various cell lineages to examine which cell lineages are upregulated by loss of DEAR1. Mammospheres were stained for cytokeratin (CK) 8/18, a luminal-specific cell surface marker, and CK5, a myoepithelial/basal-specific cell surface marker^33^. Results indicated that mammospheres contained a small population of bipotent cells in both DEAR1 control and KD clones, in the absence and presence of TGF-β (Fig. 3a, yellow in the merged figures). However, in the presence of TGF-β, *DEAR1*-KD cells showed intense staining with myoepithelial marker CK5, which was not observed for CK8/18, indicating that TGF-β promotes myoepithelial/basal-like progenitor features in *DEAR1*-KD cells (Fig. 3a, TGF-β panel). Staining for epithelial cell adhesion molecule (EpCAM) and α6 integrin (CD49f) identifies luminal and basal/myoepithelial cells from breast tissues. Mature luminal cells are characterized by EpCAM^+^/CD49f^−^ staining, EpCAM^+^/CD49f^+^ identifies luminal progenitor cells, while EpCAM^−^/CD49f^+^ identify basal progenitor cells^32^. FACS analysis using EpCAM and CD49f revealed an increased proportion of the EpCAM^−^/CD49f^+^ cell population in two *DEAR1* knockdown clones compared to control vector clones (3.82% ± 2.5 v. 0.54% ± 0.28, p = 0.04, Fig. 3b,c, and Supplementary Fig. S1). This result indicates that in *DEAR1* KD cells there is a shift away from differentiated luminal cells toward more basal progenitor and/or bipotent cell types^19^. To further explore this shift we performed FACS analysis using CD44 and CD24 surface markers to examine whether a shift from luminal to basal-like cells as characterized by lower CD24 expression and a higher CD44:CD24 ratio, a cancer stem cell index^34^, was evident. Results revealed that *DEAR1-*KD clones have a higher CD44:CD24 ratio compared to control vector clones due to a significant loss of CD24 expression (p = 0.0017 vs E6-CshR, Fig. 3d), providing further evidence of a shift of cells toward a more basal/myoepithelial profile^35^. Cumulatively, data indicate that *DEAR1* KD results in an incomplete but significant shift from luminal to more basal-like cell types, consistent with loss of DEAR1 maintenance of the luminal cell lineage.

### DEAR1 negatively regulates the master EMT/stemness transcription factor SNAI2 independently of the TGF-β-SMAD3 axis

**Figure 4:**
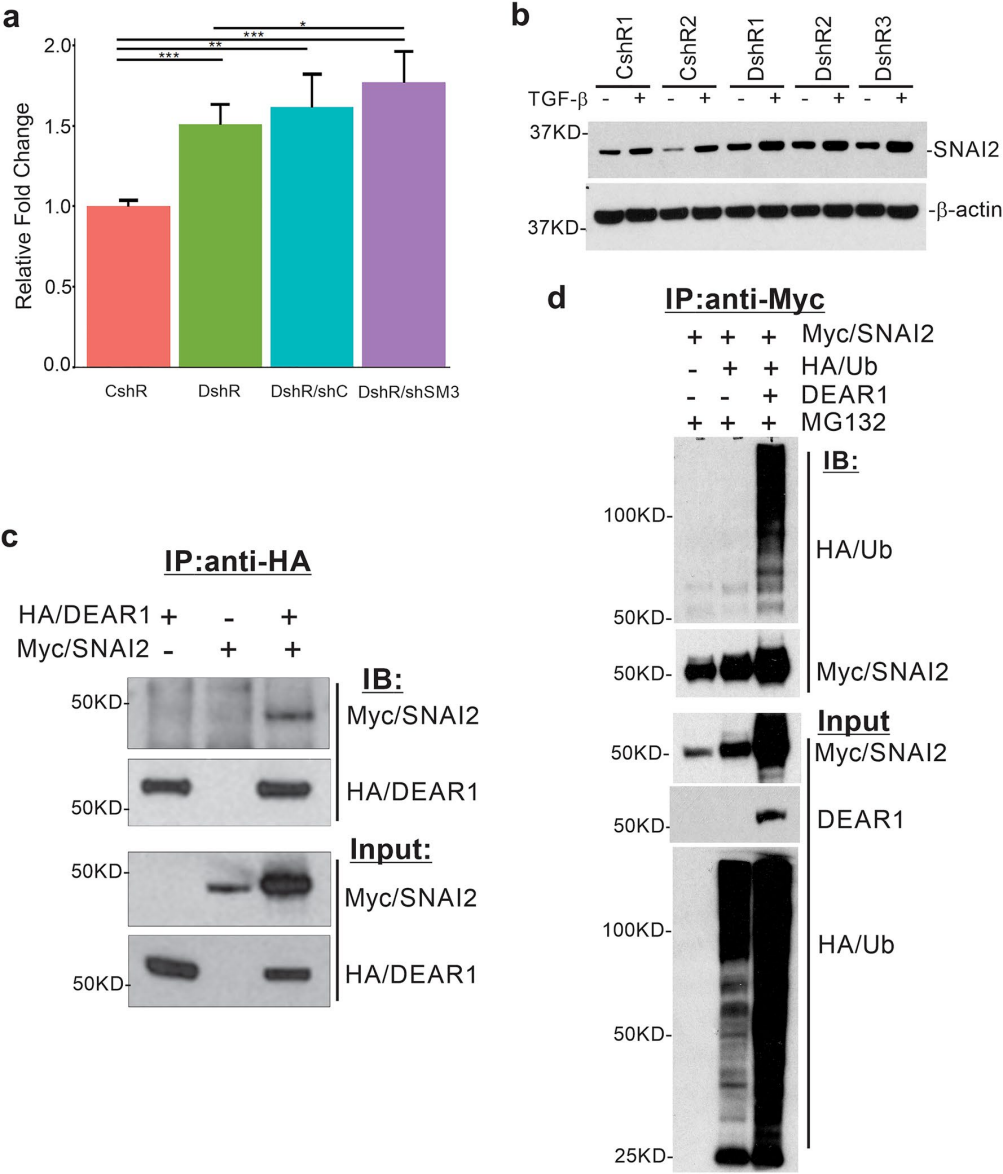
The regulation of SNAI2 by DEAR1. (**a**) Bar plots of qPCR results (mean ± SD) of *SNAI2* mRNA levels in control (CshR), *DEAR1*-KD (DshR), *DEAR1*-KD/shRCt clone (DshR/shC), and DEAR1-SMAD3 double KD (DshR-shSM3) across 2 runs. P-values were calculated using Wilcoxon Rank Sum tests. *p < 0.05–0.1, **p < 0.01, ***p < 0.001. The barplots were created using RStudio (Version 1.0.143), https://www.rstudio.com. (**b**) Western blot showing SNAI2 protein expression in control (CshR) and *DEAR1*-KD clones (DshR) with and without TGF-β treatment. Full length gels for Western blots are shown in Supplementary Fig. S2. (**c**) Co-immunoprecipitation (Co-IP) to determine possible interaction between DEAR1 and SNAI2. HEK293T cells were co-transfected with an *HA*-*DEAR1*-expressing plasmid and/or a *Myc-SNAI2* expression plasmid. After 24 h post-transfection, cell lysates were immunoprecipitated with anti-HA (rabbit) antibody. SNAI2 proteins were blotted with anti-Myc (mouse) antibody. Full length gels for Western blots are shown in Supplementary Fig. S3. (**d**) Co-IP assay to detect ubiquitination of SNAI2 by DEAR1. HEK293T cells were co-transfected with *Myc-SNAI2*, *HA*-*ubiquitin*, and *DEAR1*. Cells were lysed 48 h post-transfection. Lysates were denatured and immunoprecipitated with anti-Myc (mouse) antibody. Ubiquitin was blotted with anti-HA (Rat) antibody. Full length gels for Western blots are shown in Supplementary Fig. S4.

To investigate the molecular mechanisms underlying the shift towards expansion of basal-like cells and increased mammosphere formation upon DEAR1 loss, we focused on the master regulator of EMT and stemness *SNAI2.* SNAI2 has been shown to maintain luminal progenitor and mesenchymal phenotypes through inhibition of luminal cell differentiation and we have previously shown it to be upregulated at the mRNA level in HMECs upon knockdown of DEAR1^21,26^. Since we found DEAR1 loss of expression results in mammosphere formation independently of the TGF-β-SMAD3 axis (Fig. 1e), we then examined if DEAR1 regulates SNAI2 independently of the TGF-β-SMAD3 axis. Results indicated *SNAI2* mRNA expression levels in *DEAR1-SMAD3* double KD (DshR/shSM3) cells remained elevated relative to control (adjusted p < 0.001,Fig. 4a), similar to the *DEAR1* knockdown alone (DshR), and had no significant difference with DshR SM3-control (DshR/shC) (p = 0.31, Fig. 4a). These results demonstrate that loss of DEAR1 can confer upregulation of *SNAI2* mRNA levels independently of SMAD3 signaling. At the protein level, SNAI2 expression in *DEAR1*-KD HMECs was elevated compared to control vector clones, with enhanced expression in the presence of TGF-β (Fig. 4b and Supplementary Fig. S2). Cumulative results indicate that DEAR1 regulates SNAI2 expression both independently and through the TGF-β-SMAD3 axis.

### E3 Ligase DEAR1 binds to and ubiquitinates SNAI2.

We further investigated if DEAR1 directly regulates SNAI2 at the protein level. Co-immunoprecipitation in HEK293T cells using Myc-SNAI2 and HA-DEAR1 expression constructs demonstrated that DEAR1 interacts with SNAI2 (Fig. 4c and Supplementary Fig. S3). We then co-transfected *Myc-SNAI2*, *HA-ubiquitin*, and pcDNA-*DEAR1* into HEK293T cells to investigate whether DEAR1 promotes the ubiquitination of SNAI2. Myc-SNAI2 pulldown by anti-Myc was observed as well as polyubiquitination of SNAI2 was detected using anti-HA tag antibody. Results indicated that cotransfection of DEAR1 promoted SNAI2 polyubiquitination (Fig. 4d and Supplementary Fig. S4). These results demonstrate that DEAR1 functions independently of the canonical TGF-β pathway to regulate SNAI2 expression and activity by promoting SNAI2 ubiquitination, thereby also regulating SNAI2 expression at the protein level in addition to the transcript level.

### Loss of DEAR1 expression predicts early age of onset of TNBC and shorter time to metastasis

Our previous work studied a cohort of 123 early onset breast cancers, which identified that DEAR1 was a strong predictor of local recurrence-free progression, as well as correlated with the triple negative phenotype^27^. As many of these tumors fall under the basal subtype, coupled with our results showing the expansion of progenitor and basal-like cells due to lack of inhibition of SNAI2 upon DEAR1 loss, we interrogated several publicly available datasets of TNBC and basal-like breast cancers (BLBC) for DEAR1 expression trends. Utilizing bc-GenExMiner v4.0^36,37^, we found a significant trend in *DEAR1* mRNA downregulation in basal-like tumors compared to other subtypes (p < 0.0001, Supplementary Fig. S5a), as well as in the TNBC subgroup (p < 0.0001, Supplementary Fig. S5b).

**Figure 5:**
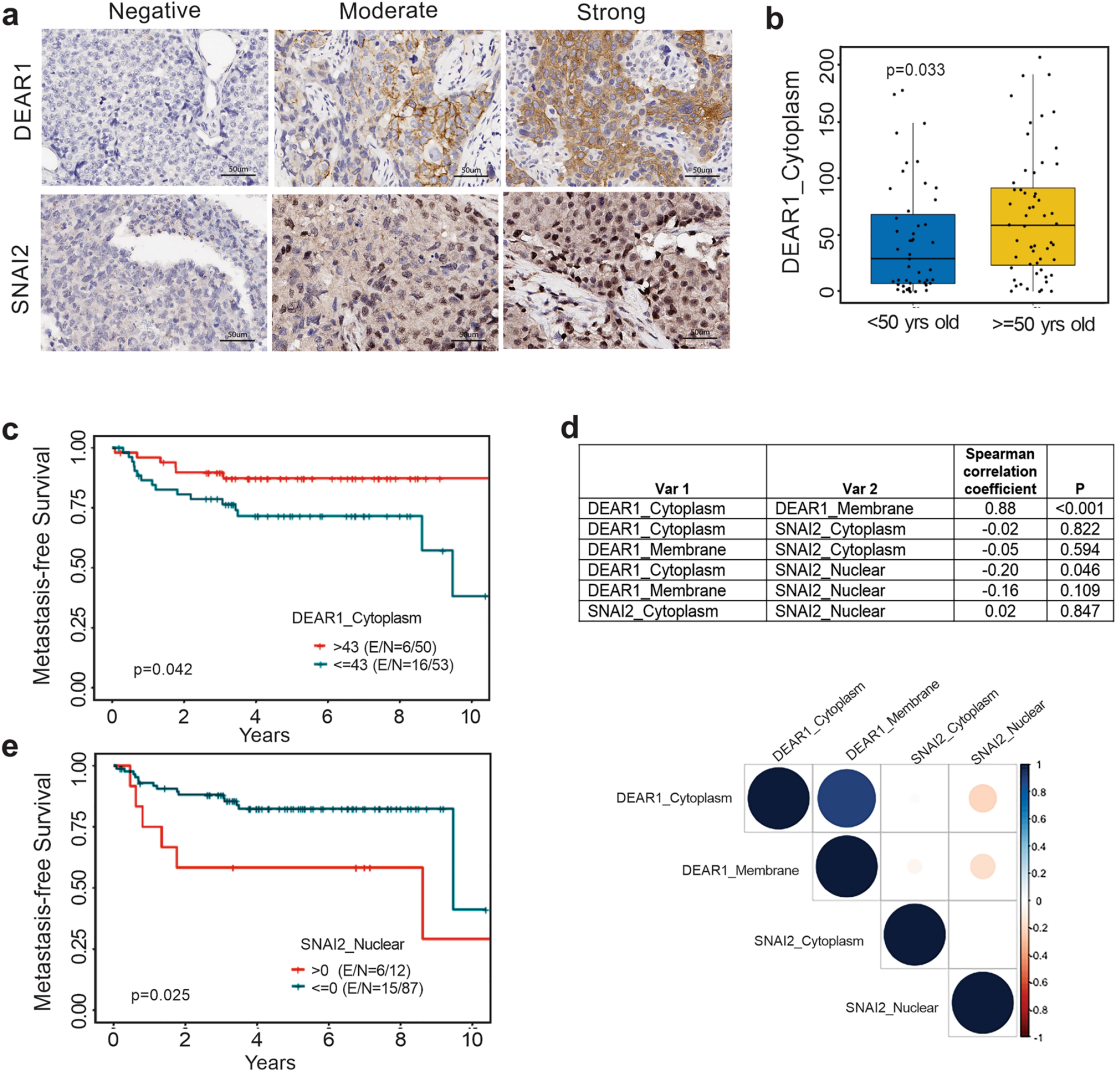
DEAR1 and SNAI2 expression analysis in a TNBC TMA cohort. (**a**). Representative images of DEAR1 and SNAI2 IHC in TNBC TMA analysis (Scale bars equal 50 µm). (**b**) Boxplot of cytoplasmic DEAR1 expression in groups of patients under (blue) or over (yellow) the age of 50 years. (**c**) Kaplan–Meier analysis of metastasis-free survival in DEAR1 groups dichotomized by the median of cytoplasmic DEAR1 expression (High expression = red, low = blue). (**d**) Spearman’s correlations of DEAR1 and SNAI2 expression across different cellular fractions. Spearman’s correlation was performed using R Statistical Software (version 3.4.2; R Foundation for Statistical Computing, Vienna, Austria). (**e**) Kaplan–Meier analysis of metastasis-free survival in SNAI2 groups dichotomized by presence (red) or absence (teal) of nuclear SNAI2 expression. Both (**c**) and (**e**) were analyzed by the log-rank test.

Since we observed a significant reduction of *DEAR1* expression in BLBCs and TNBCs, we further investigated the potential of utilizing DEAR1 expression to predict TNBC patient outcomes. In a tissue microarray containing 103 TNBC samples (for patient characteristics, see Supplementary Table S2), we stained for DEAR1 and SNAI2. As illustrated in Fig. 5a, DEAR1 is mainly expressed in the cytoplasm but can also be detected in the membrane, while SNAI2 expression is located in the cytoplasm and nucleus. A statistically significant association between age at diagnosis and DEAR1 cytoplasmic expression was detected. When using an age of 50 as the cutoff, older patients had higher DEAR1 cytoplasm expression compared to younger patients (p = 0.033, Fig. 5b). Low DEAR1 cytoplasmic expression, when dichotomized by the median value, was also associated with a shorter time to metastasis (p = 0.042, Fig. 5c) in the TNBC patients (see Supplementary Table S3). These data indicate that DEAR1 expression could be useful in stratifying TNBC for risk of developing early-onset TNBC and aggressive metastasis. In line with our results showing DEAR1-mediated SNAI2 ubiquitination (Fig. 4d), we found that DEAR1 cytoplasmic and SNAI2 nuclear levels were negatively correlated in the TMA (Fig. 5d) and the presence of SNAI2 nuclear expression was significantly associated with a shorter time to metastasis (p = 0.025, Fig. 5e).

## Discussion

**Figure 6:**
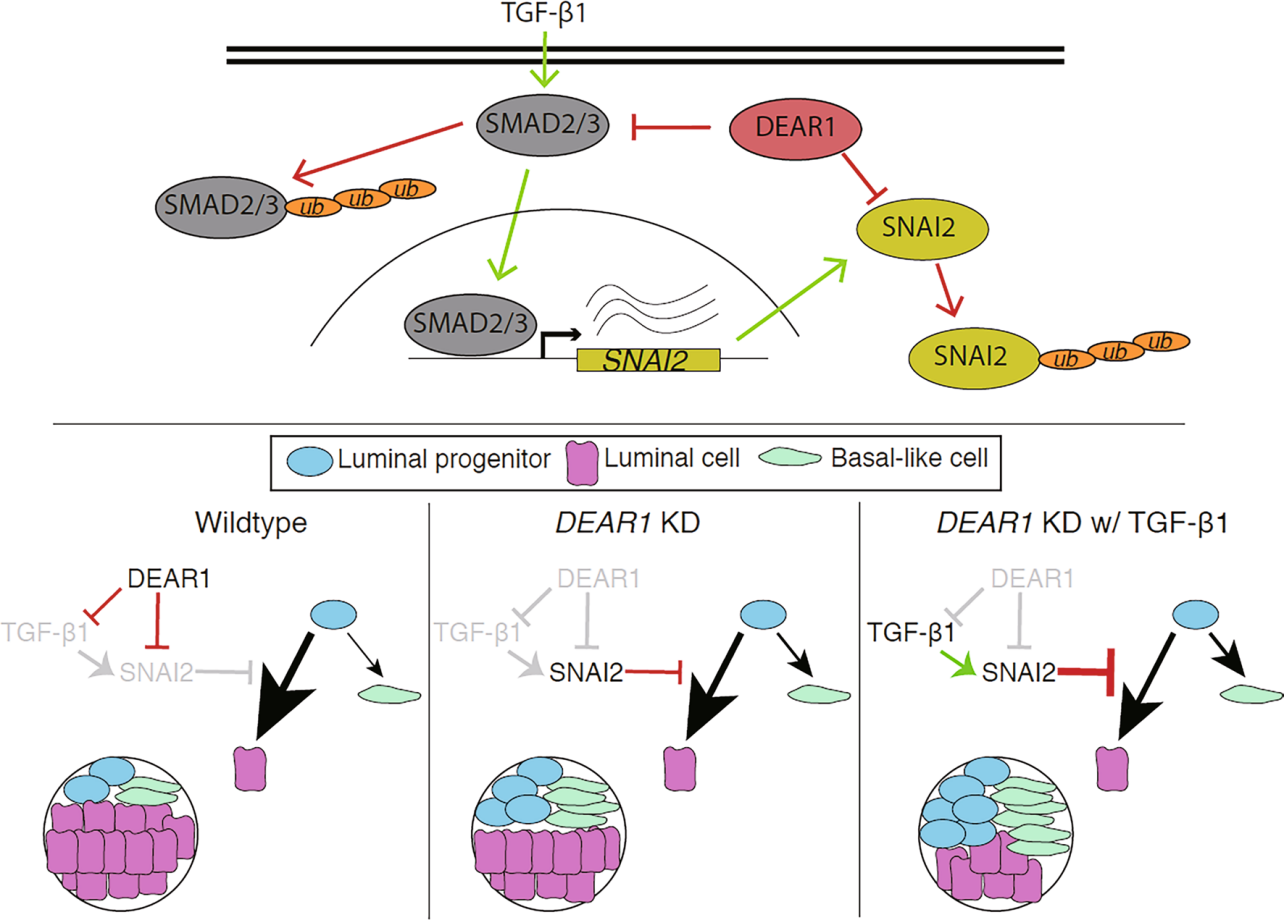
Simplified graphical model of DEAR1 regulation of luminal differentiation via TGF-β dependent and indepdendent mechanisms of SNAI2 inhibition. Top: DEAR1 inhibits SNAI2 (red pathway) by direct binding and uibiquitination, as well as by blocking *SNAI2* transcription by binding and ubiquitinating upstream activator SMAD3 that is activated by TGF-β (green pathway). Bottom: Shifts in luminal differentiation dynamics or state occupancies (as depicted by arrow sizes, where larger size indicates higher likelihood) of luminal progenitors (blue), toward incomplete basal-like (green) or mature luminal cells (pink), depending on the presence (black) or absence (gray) of DEAR1,SNAI2, and TGF-β. The figure was created using Adobe Illustrator 2020 (Version 24.2.3), https://www.adobe.com/products/illustrator.html.

Our work highlights the role of DEAR1 in maintaining proper luminal differentiation of human mammary epithelial cells, complementing our previous work showing failure of lumen formation and acinar morphogenesis upon knockdown of *DEAR1*^27^. We extend these findings to document herein that loss of DEAR1 significantly upregulates mammosphere formation through enhancing progenitor cell activity, reprogramming the basal/myoepithelial profile, and upregulating the master EMT and stem cell transcription factor SNAI2. SNAI2 has been shown to maintain a luminal progenitor fate by inhibiting luminal differentiation in normal HMECs^21^. Results demonstrate that DEAR1 acts as an upstream inhibitor of this process by directly binding to and ubiquitinating SNAI2. The prolonged time spent in a plastic luminal progenitor-like state (i.e. ALDH-expressing cells) in the absence of DEAR1 allows increased opportunity for the spontaneous generation of a mesenchymal-like CD44^high^/CD24^low^ state, which reflects an altered or incomplete myoepithelial differentiation^21^, consistent with our results (Fig. 6). Importantly, this subpopulation is associated with increased mammosphere formation due to its survival ability in anchorage-independent conditions, invasive properties at the tumor front, and is enriched in the BLBC subtype^13,38^. Therefore, loss of DEAR1 disrupts normal mammary epithelial homeostasis, leading to the generation of greater quantities of invasive mesenchymal-like cells with basal signatures^39^. This may be an early event in the tumorigenesis of basal-like breast cancers and provides an explanation for our previous observation of lower DEAR1 expression correlating with the triple-negative phenotype^27^, as well as our current association between lower DEAR1 expression with earlier age of onset and time to metastasis in a large TNBC cohort.

Our current results also indicate that the generation of basal/myoepithelial-like cells from luminal progenitor-like precursors, which occurs through EMT effectors such as SNAI2^21^, is further amplified upon exposure to TGF-β in the absence of DEAR1. This is consistent with our previous work showing DEAR1 as a negative regulator of TGF-β-driven EMT and migration in HMEC cells, and by inhibiting SMAD3 and its downstream effectors such as SNAI2^26^. Our results herein document compelling evidence that DEAR1 controls luminal differentiation through a TGF-β independent axis with DEAR1 functioning as an E3 ligase to bind to and ubiquitinate SNAI2 independent of TGF-β. In addition to repressing the luminal cell state, SNAI2 has been shown to induce mammosphere formation and an expansion of the basal progenitor state^28,40–43^. Thus, DEAR1 regulates luminal cell fate and mammosphere formation through binding to and ubiquitinating SNAI2. Taken together, these data and our previous work indicate DEAR1 functions to inhibit EMT and stemness through multiple mechanisms which now includes limiting SNAI2 expression at both the transcriptional and posttranslational levels (Fig. 6).

Herein we document that DEAR1 governs epithelial plasticity by maintaining luminal differentiation as an ubiquitin ligase for SNAI2, even in the presence of exogenous stimulus from TGF-β. Loss of DEAR1 significantly upregulates mammosphere formation and increases the progenitor cell activity and the basal/myoepithelial state. In addition, microenvironmental sources of TGF-β contribute to the aggressive and metastatic nature of TNBC by enhancing SNAI2’s activity to further increase the plasticity of progenitors and subsequent numbers of invasive mesenchymal-like cancer cells (Fig. 6). In addition to the SMAD3 pathway, TGF-β also induces SNAI2 via non-SMAD pathways to drive metastasis via major oncogenic signaling such as PI3K/Akt/mTOR and MAPK (ERK, p38, or JNK) pathways^44–47^. Interestingly, DEAR1/TRIM62 has been reported to suppress tumor metastasis through inhibiting MAPK/JNK-induced SNAI2 expression^48^. This report then, in concert with our published and data reported herein, indicate that DEAR1 is a critical regulator of a major cytokine pathway, blocking TGF-β signaling and preventing the induction of SNAI2- driven increase basal-mesenchymal-cell populations, as well as binds directly to SNAI2 to block oncogenic signaling pathways, including reported inhibition of metastatic signaling cascade through SNAI2-MAPK/JNK pathways.

Additionally, results suggest possible important translational potential. We have shown a a significant negative correlation between DEAR1 and SNAI2 in a large TNBC cohort. Furthermore, a significant association was observed between lower DEAR1 expression and earlier age of onset and time to metastasis. Hence, DEAR1 may serve as a valuable prognostic and predictive biomarker for highly aggressive forms of TNBC. Our results furthermore suggest impact for therapuetic targeting of TNBC patients. The expanded population of CD44^high^/CD24^low^ cells we observed upon DEAR1 loss has been shown to contribute to resistance to multiple chemotherapeutic drugs^14,49^. Moreover, the cell-state plasticity and associated cell-state heterogeneity that contributes to drug resistance in TNBC and BLBC, appears to be targetable by co-treatment with the PI3K/mTOR inhibitor BEZ235 and the BET inhibitor JQ1 in model systems^4^. Therefore, DEAR1 expression could be a valuable biomarker for stratifying patients for such therapies. Future work elucidating the pathways upstream, as well as others downstream of DEAR1 will aid in identifying additional therapeutic targets aimed at cell plasticity and controlled by DEAR1.

## Materials and methods

### Cell culture

76N-E6 HMEC clones were grown in D-medium as previously described ^26^. HEK 293 T cells purchased from the MD Anderson characterized cell line core (CCLC) were grown in DMEM (Corning, MT10017CV) and 5% FBS (Sigma, F8192). MCF10A cells were cultured in medium as previously described^26^. Cell lines were authenticated by STR profiling and all cell lines are routinely checked for mycoplasma contamination using the Lonza MycoAlert kit (LT07-118).

### Western blot analysis

To obtain whole cell lysates, cells were harvested using 1× SDS sample buffer with 50 mM DTT. Equal amount of protein were loaded into a 4–12% SDS-PAGE gradient gel (ThermoFisher Scientific, NW04120BOX), and transferred onto a nitrocellulose membrane (BioRad, 1620094). SNAI2/SLUG (C19G7, #9585) was purchased from Cell Signaling (Danvers, MA, United States). Westerns were normalized using β-actin (A5441) purchased from Sigma (Saint Louis, MO, United States).

### Plasmids

The pcDNA-DEAR1 and pCMV-HA-DEAR1 constructs were generated as previously described^26^. *SNAI2* cDNA was amplified from the pEGFP-C2-SNAI2 vector (kindly provided by Dr. Kaname Kawajiri, Saitama Cancer Center, Saitama, Japan) using PCR and ligated into a pcDNA-6Myc vector digested with EcoRI and XhoI. pMT123-8x-HA-Ubiquitin was kindly provided by Dr. Dirk Bohmann (Department of Biomedical Genetics, University of Rochester Medical Center, Rochester, New York).

### Mammosphere assay

HMECs (MCF10A or 76N-E6) were grown in 2D culture in normal growing conditions. The mammosphere assay was followed as previously described with some modifications. The mammosphere medium contained Mammary Epithelial Growth Medium Complete (LONZA, CC-3051, without BPE), 20 ng/mL bFGF (Corning, 47743-574), 10 ng/mL EGF (Sigma, E9644), 4 µg/mL heparin (Sigma, H3149-50KU), and 1% methylcellulose (Sigma, M0430-100G). When required, cells were pre-treated with 4 ng/mL TGF-β1 for 48 h prior to seeding cells in mammosphere growing conditions. Cells were seeded into 4–8 wells at 1 × 10^3^ cells per well in flat bottom ultra-low attachment 96-well plates (Corning, 3474) and propagated according to published protocol^50^. Mammospheres larger than 50 µm were counted and imaged using a Zeiss light microscope at 10× magnification on Day 14. Secondary mammospheres generated by harvesting primary mammospheres, were chemically dissociated with 1× TrypLE Express, and mechanically dissociated with a pipette to achieve a single cell suspension. Cells were re-counted, seeded at 500 cells/well and monitored for growth. For these experiments, 2 stable control vector (CshR) clones and 3 *DEAR1*-KD (DshR) stable clones were used and experiments were performed in triplicate.

### Fluorescence activated cell sorting (FACS)

Control clones and *DEAR1*-KD 76N-E6 clones were grown in 2D culture and stained for FACS using EpCAM-PE (eBioscience, 12-9326-41), CD49f-APC (eBioscience, 17-0495-80), CD24-PE (BD Biosciences, 555428), and CD44-FITC (BD Biosciences, 555478) according to the manufacturer’s Staining Cell Surface Antigens for Flow Cytometry protocol. Plated cells were trypsinized, counted, and resuspended in Flow Cytometry Staining Buffer for a final concentration of 1 × 10^7^ cells/mL. 50 µL of the cell suspension was incubated with primary antibody so that the final volume was 100 µL per sample. The cells incubated for 30 min in the dark at 4 °C, washed, and analyzed. For these experiments, 2 stable control vector (CshR) clones and 2 *DEAR1*-KD (DshR) stable clones were used experiments were performed in triplicate.

### Immunofluorescence

Whole mammospheres were collected and resuspended in 100 µL of 1× PBS for cytospin centrifugation onto slides using Shandon Cytospin 4 (Thermo Scientific, 1000 rpm, 8 min). Slides were stained using standard immunofluorescence staining protocol using 0.3% Triton X-100 in PBS for permeabilization and blocked with anti-Goat serum for one hour at 4 °C. Anti-CK8/18 (rabbit, Abcam, cat#: ab53280, 1:100), anti-CK5 (mouse, Pierce, cat#: MA5-17057, 1:100), and ALDH1A1 (rabbit, Abcam, cat#: ab52492, 1:100) were used to examine co-expression of luminal and basal cytokeratins and a marker of stem/progenitor cells. Secondary antibodies goat anti-mouse (Alexa 488) and goat anti-rabbit (Alexa 555) were used. Nuclei were stained with DAPI (Sigma, 236276). Slides were treated with ProLong Gold anti-fade fluorescent mounting agent (ThermoFisher Scientific, P10144). Fluorescent images were taken using OLYMPUS cellSens Software.

### Primers

To amplify *SNAI2* from the pEGFP-C2 backbone via PCR, forward and reverse primers were designed to include EcoRI and XhoI restriction enzyme cut sites, respectively, for ligation into the pcDNA-6Myc backbone (FW primer, 5′-TAACGAGAATTCATGCCGCGCTCCTTC-3′; RV primer, 5′-TCGTTACTCGAGTCAGTGTGCTACACAGCA-3′).

### Co-immunoprecipitation and ubiquitination assays

For co-immunoprecipitation (Co-IP) assays, HEK293T cells were transfected with Myc-SNAI2 and/or HA-DEAR1 plasmids in a 6 cm plate using Mirus TransIT-LT1 transfection reagent (Mirus, MIR 2300) for 24 h. Cells were treated with MG-132 proteasome inhibitor (Calbiochem, 133407-82-6) for 2 h, before harvest. Cells were lysed with M-PER lysis buffer (ThermoFisher Scientific, 78501) and incubated with pull-down antibody overnight at 4 °C. Then, lysates were incubated with protein A/G agarose beads (Santa Cruz, sc-2003) for 2 h at 4 °C followed by three 5-min washes with RIPA lysis buffer. Proteins were eluted from beads in 30 µL of 2× SDS sample buffer for western blot analysis. Rabbit polyclonal anti-HA (H6908) or anti-Myc (M4439) from Sigma were used for pull-down and Mouse monoclonal anti-HA (H3663) from Sigma or anti-Myc (#562) from MBL International Corporation were used for immunoblotting. For in vivo ubiquitination assays, HEK293T cells were transfected with Myc-SNAI2, 8x-HA-Ub, and/or DEAR1 plasmids in a 10 cm plate. At 20 h after transfection, cells were treated with MG132 20 μmol/L for 4 h and harvested. Cells were lysed with 1× SDS-lysis buffer, denatured by heating at 95 °C for 10 min and then diluted with 0.5% NP-40 buffer and used for further analysis.

### Quantitative real-time PCR (qRT-PCR)

Cells were treated with 4 ng/mL TGF-β1 (EMD Millipore, 616450-1UG) for 3 and 40 h, RNA extracted and reverse transcribed using the High Capacity cDNA Reverse Transcription kit (Applied Biosystems, 4368814). Real time quantitative PCR was performed using TaqMan^®^ Universal Master Mix II, no UNG (Applied Biosystems, 4440047) and TaqMan^®^ probes for TRIM62 (Hs00217089_m1), SNAI2 (Hs00950344_m1), ZEB1 (Hs00232783_m1), ZEB2 (Hs00207691_m1), and GAPDH (Hs02786624_g1) in triplicate and in MicroAmp optical 384-well plates (Applied Biosystems, 4309849).

### Breast cancer tissue microarray (TMA) construction and patient cohort

The study was approved by the institutional review board of MD Anderson Cancer Center (IRB protocol number: PA14-0318), and all methods were carried out in accordance with approved guidelines. Informed consent was obtained from all patients to use residual tissue for research. Archival, formalin-fixed and paraffin-embedded (FFPE) material from surgically resected breast cancer specimens were obtained from the Breast Tumor Bank at M. D. Anderson Cancer Center from 2001 to 2013. Tumor tissues were histologically examined, classified using the World Health Organization (WHO) classification of Breast Tumors and selected for TMA construction, after which TMAs were prepared using triplicate 1-mm-diameter cores per tumor.

### Patient characteristics

Detailed clinical and pathologic information, including demographic, pathologic TNM staging, overall survival, and time of recurrence was collected according to Institutional Review Board guidelines with informed consent. A total of 103 TNBC patients were included in the analysis, and the average age was 54 (range 26, 80).

### Immunohistochemistry (IHC) for breast cancer tissue microarray (TMA)

DEAR1 staining was performed as previously described^26^. SNAI2 staining utilized an antibody purchased from Santa Cruz, Biotechnology (A-7, SC-166476, 1:50 dilution) on Leica BOND-MAX. DEAR1 and SNAI2 expression were evaluated using Aperio to determine H-scores. Both DEAR1 and SNAI2 expression in the cytoplasm and nucleus of breast tumor cells on the TMA were evaluated separately using a standard microscope approach. Expression was scored by staining intensity 0 (no staining), 1 + (weak staining), 2 + (moderate staining), or 3 + (strong staining) and the percentage of cells with postiive IHC expression as well as an H Score was obtained (0–300).

### Statistics

All values for in vitro analyses are expressed as mean ± standard deviation (SD) and represent three independent trials. Unless otherwise specified, comparisons between groups were performed using a two-tailed Student t-test. In the case of multiple comparisons, p-values were corrected using the Benjamini and Hochberg method. For analyses involving the TMA, Wilcoxon or Kruskal–Wallis rank sum tests were used to assess the difference of biomarkers between groups. Overall survival and time to metastasis were defined as the time between date of surgery and date of death or date of metastasis, respectively. Their survival functions were estimated using the Kaplan–Meier method and log-rank test was used to compare the survival between groups. All tests were 2-sided and p-values < 0.05 was considered statistically significant. R version 3.4.2 was used to perform statistical analyses. Asterisks represent: *p < 0.05–0.1, **p < 0.01, ***p < 0.001, and ****p < 0.0001.

## Supplementary Information

Supplementary Information.
