# Supplementary material for: Tumor suppressor DEAR1 regulates mammary epithelial cell fate and predicts early onset and metastasis in triple negative breast cancer
Source: Sci Rep. 2022 Nov 14;12:19504. doi: 10.1038/s41598-022-22417-4 (PMC9663828; doi:10.1038/s41598-022-22417-4)
Supplement: Supplementary file 1 — Supplementary Information. [file 41598_2022_22417_MOESM1_ESM.pdf]

Tumor suppressor DEAR1 regulates mammary epithelial cell fate and predicts early onset and metastasis in triple negative breast cancer

Uyen Q. Le, Nanyue Chen, Seetharaman Balasenthil, Eugene Lurie, Fei Yang, Suyu Liu, Laura Rubin, Luisa Maren Solis Soto, Maria Gabriela Raso, Harsh Batra, Aysegul A. Sahin, Ignacio I. Wistuba and Ann McNeill Killary

Supplementary information

Figure S1: The isotype control of CD49f and EpCAM for figure 3b

Figure S2: Whole western blots for Figure 4b.

Figure S3: Whole western blots for Figure 4c.

Figure S4: Whole western blots for Figure 4d.

Figure S5: DEAR1 expression is significantly reduced in basal-like breast cancer.

Table S1: Loss of DEAR1 expression does not confer HMECs with self renewal capabilities.

Table S2: TMA patient demographics.

Table S3: Summary of metastasis-free survival between marker groups in triple negative breast cancer group.

a

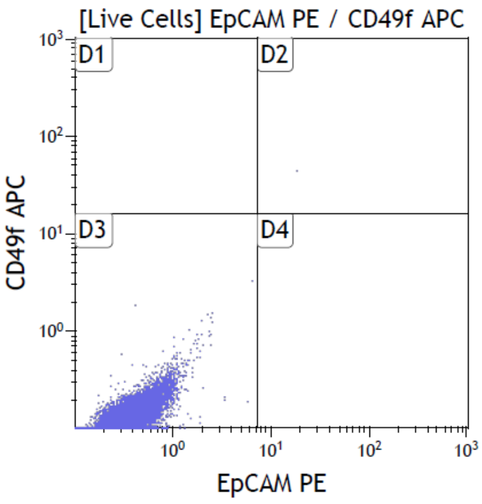

b

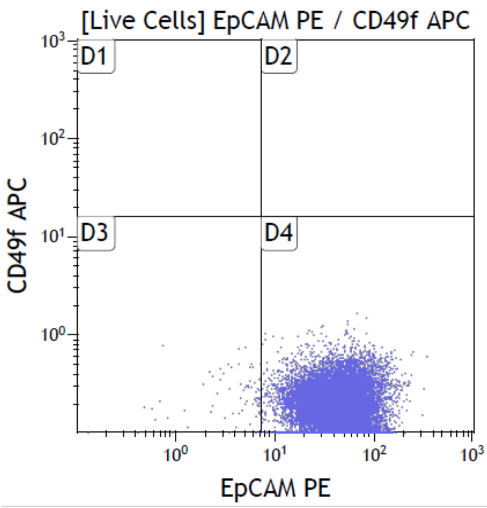

c

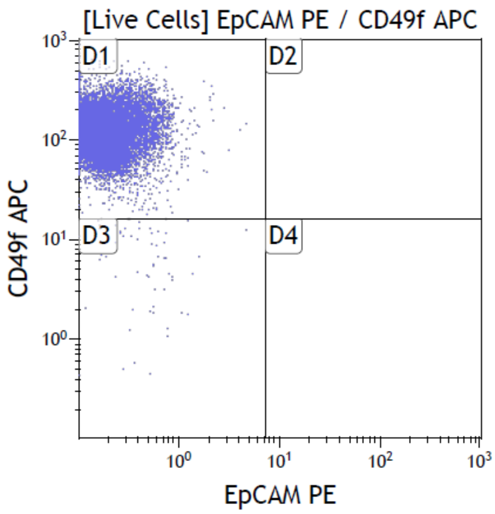

Fig. S1: The isotope control of CD49f and EpCAM for figure 3b. a) Negative control without any staining; **b)** Stain with EpCAM alone; c) Stain with CD49f alone.

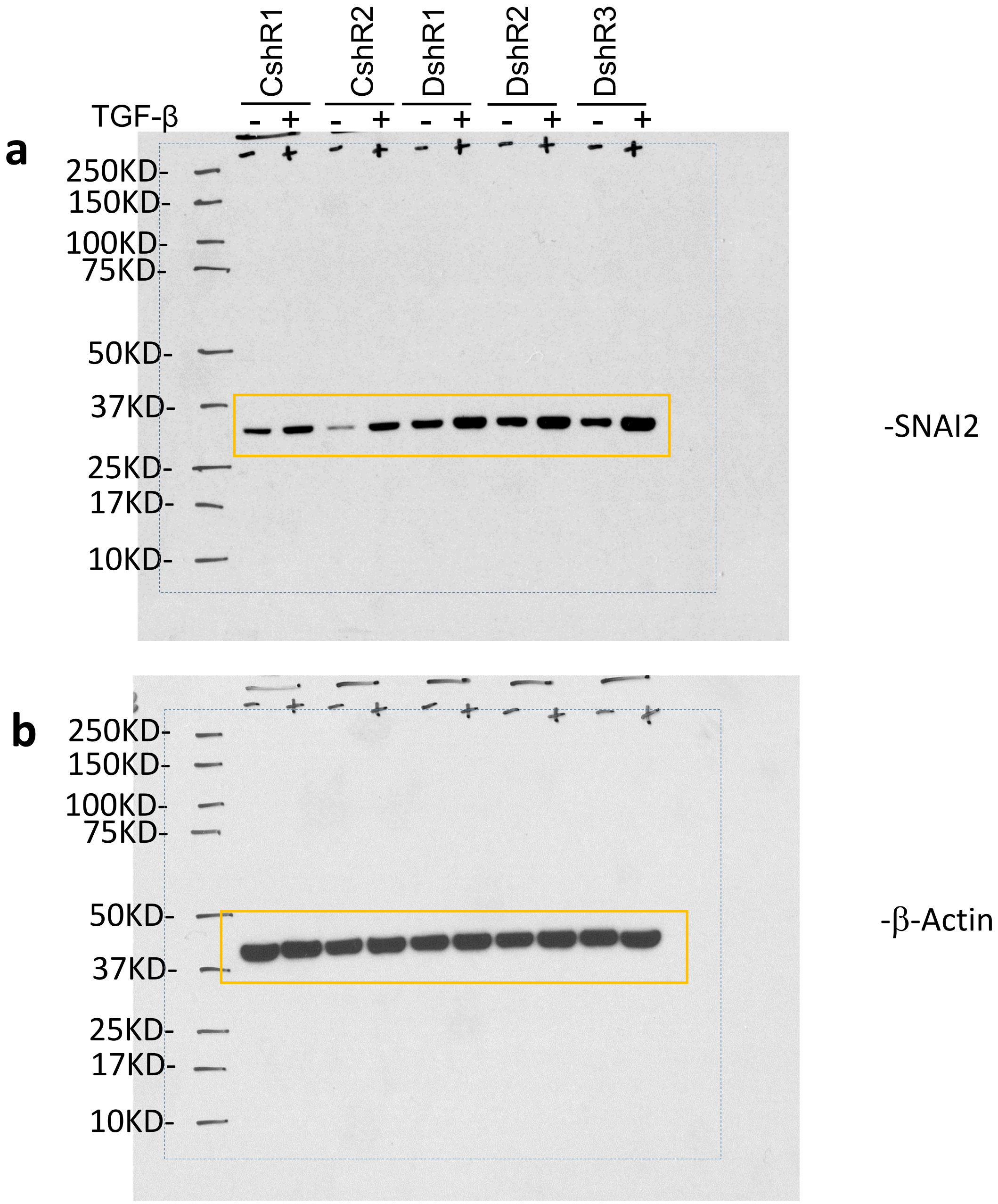

Fig. S2: Whole western blots for Figure 4B. **a)** the blot to show SNAI2; **b)** the same gel of a) to show b-actin.

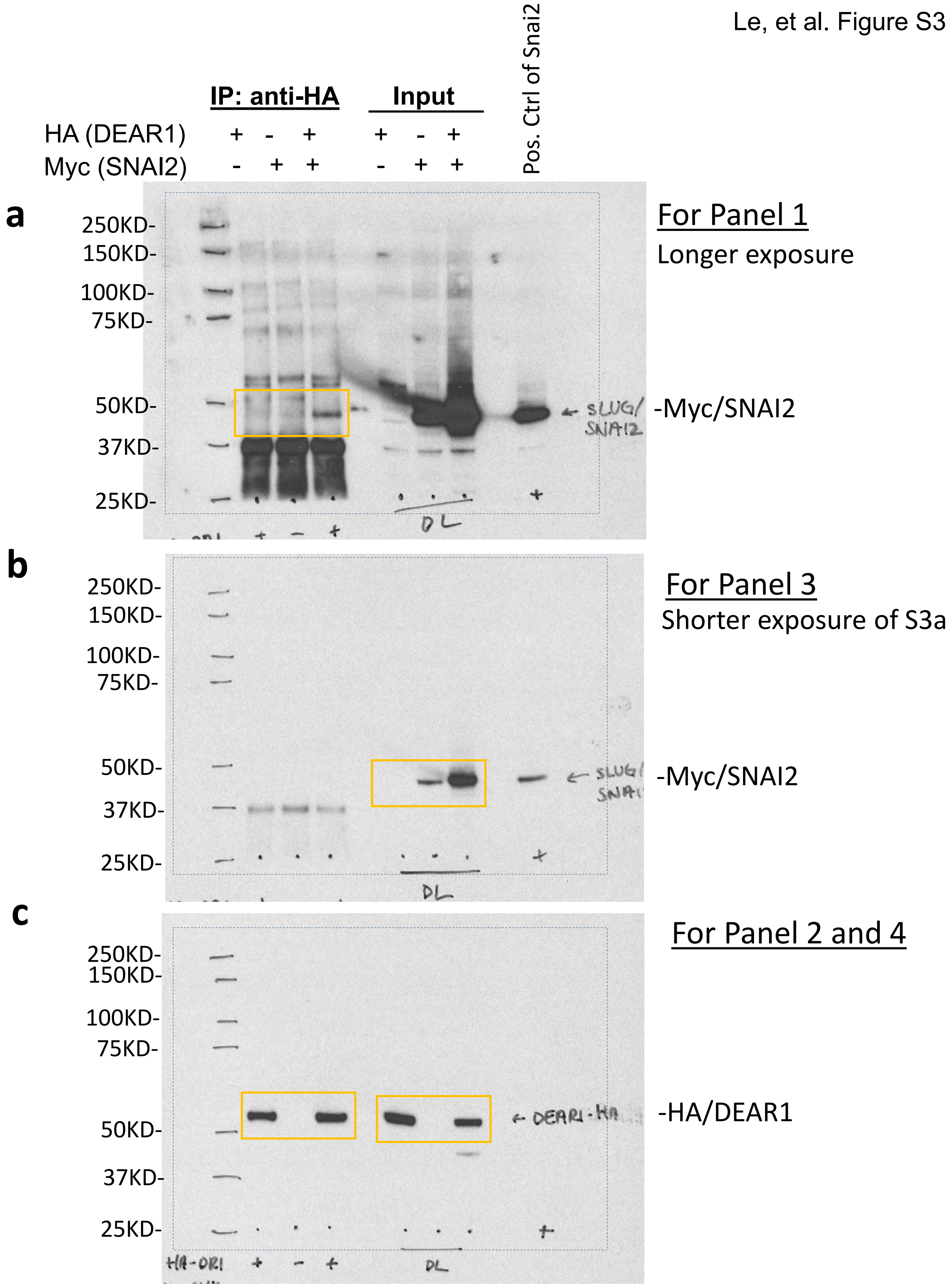

Fig. S3: Whole western blots for Figure 4C. **a)** longer exposure of the blot to show Myc/SNAI2 in left panel for IP; **b)** shorter exposure of same blot of a) to show Myc/SNAI2 in right panel for input; **c)** same gel of a) to show HA/DEAR1.

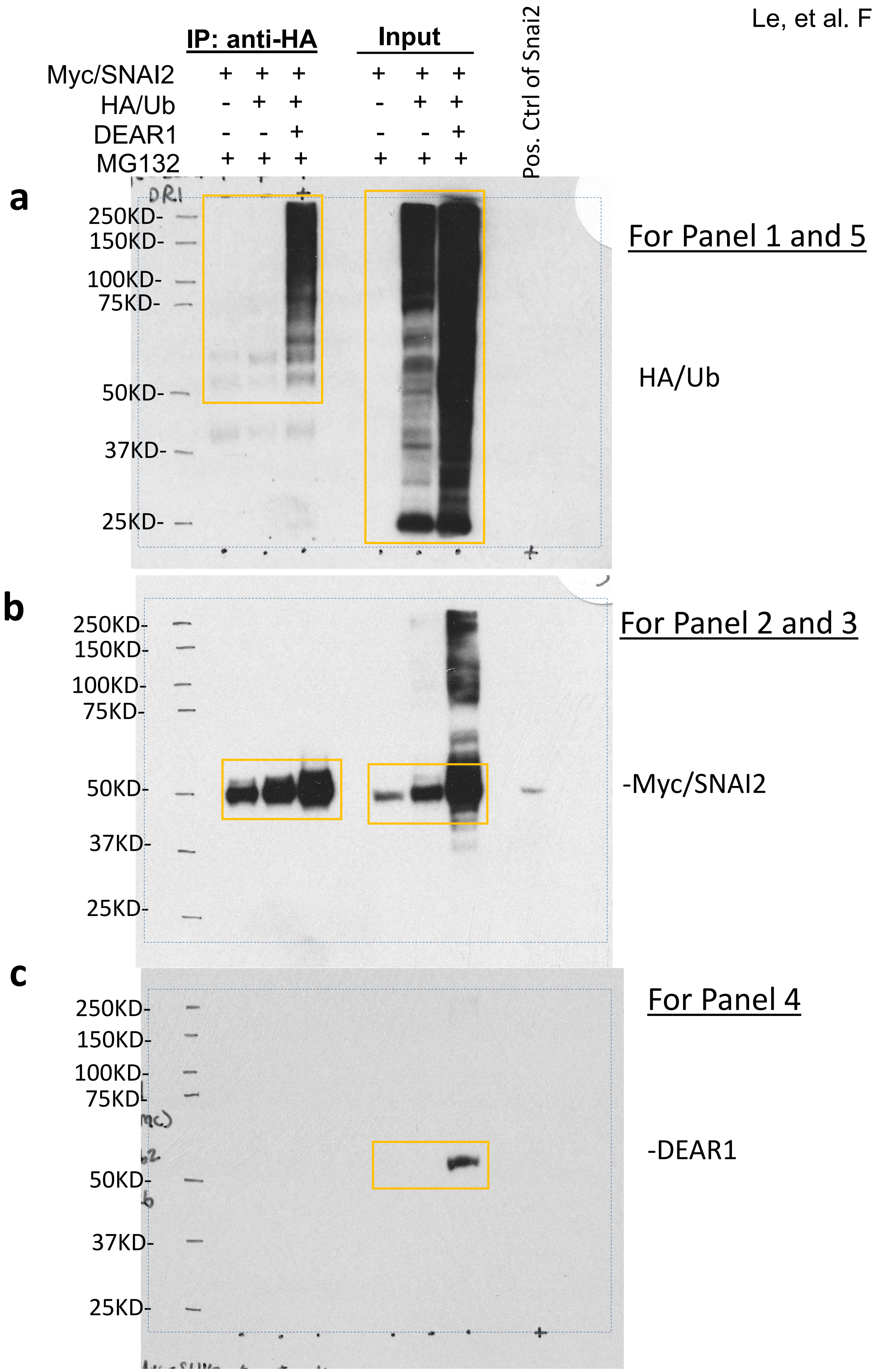

Fig. S4: Whole western blots for Figure 4D. a) the blot to show HA/Ub in left panel for IP and right panel for input; **b)** the same gel of a) to show Myc/SNAI2 in left panel for IP and right panel for Input; **c)** same gel of a) to show DEAR1.

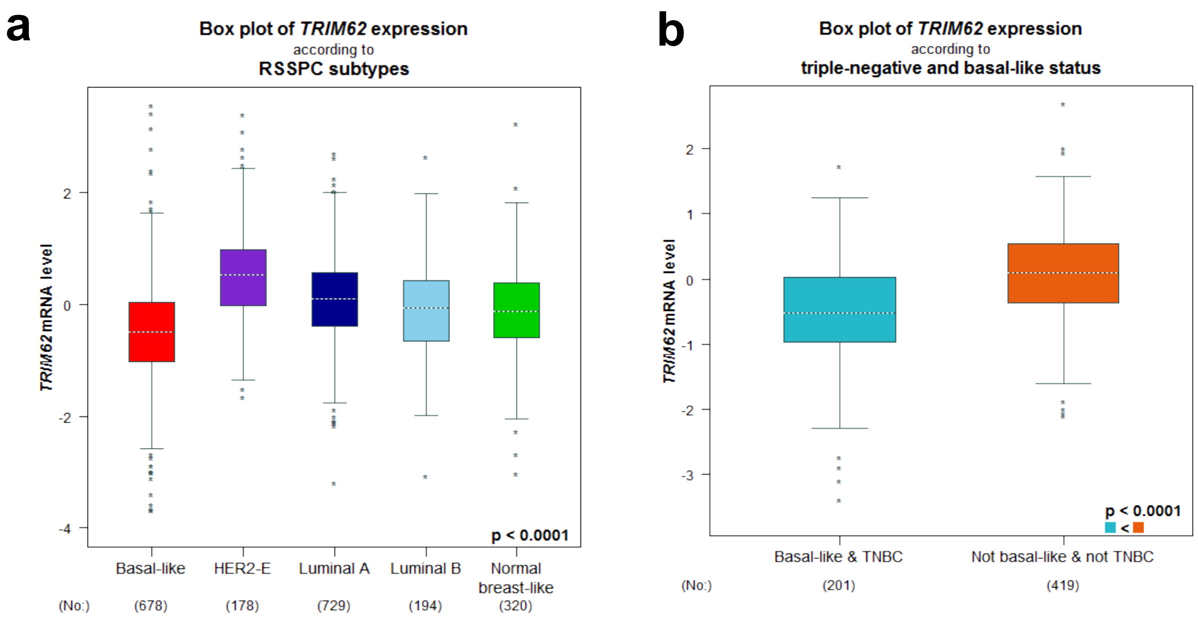

Fig. S5: **DEAR1 expression is significantly reduced in basal-like breast cancer.** *In silico* data indicates that *DEAR1* mRNA expression is significantly reduced **a)** in basal-like breast cancer compared to other subtypes (p<0.0001) and **b)** in the TNBC subgroup (p<0.0001). The data were analyzed with bc-GenExMiner v4.0. The URL is: http://bcgenex.ico.unicancer.fr/BC-GEM/GEM-Accueil.php?js=1

Table S1: **Loss of DEAR1 expression does not confer HMECs with self renewal capabilities.**

No TGF-β

**HMEC clone**	**Passage**	**MFE**	**Fold Δ**
CshR1	1	0.325%	0.69
	2	0.267%	
CshR2	1	0.225%	1.47
	2	0.33%	
DshR1	1	1.55%	1.68
	2	2.6%	
DshR2	1	0.675%	0.1
	2	0.067%	
DshR3	1	1.275%	0
	2	0%	

TGF-β

**HMEC clone**	**Passage**	**MFE**	**Fold Δ**
CshR1	1	0.325%	0.62
	2	0.2%	
CshR2	1	0.075%	0
	2	0%	
DshR1	1	1.85%	0.76
	2	1.4%	
DshR2	1	1.5%	0.53
	2	0.8%	
DshR3	1	2.7%	0.37
	2	1.0%	

Note: Mammosphere forming efficiency (MFE) is calculated as a percentage of the number of mammospheres larger than 50uM formed out of the total number of single cells seeded per well. In both the absence and presence of TGF-β, loss of DEAR1 expression in HMECs does not result in a significant increase in MFE after passaging primary mammospheres.

Table S2: **TMA patient demographics**

Variable	level	N	%
Stage	Unknown	6	5.83
	IA	42	40.78
	IB	0	0.00
	IIA	30	29.13
	IIB	11	10.68
	IIIA	9	8.74
	IIIB	1	0.97
	IIIC	2	1.94
	IV	2	1.94
Status	Alive	79	76.70
	Dead	24	23.30
Metastasis	No	81	78.64
	Yes	22	21.36
Recurrence	Unknown	6	5.83
	No	94	91.26
	Yes	3	2.91
Race	Asian	5	4.85
	Black	15	14.56
	Hispanic	15	14.56
	White	66	64.08
	Unknown	2	1.94
TNM classification			
T	T1	50	51.55
	T2	37	38.14
	T3	8	8.25
	T4	2	2.06
N	N (Unknown)	8	7.77
	N0	65	63.11
	N1	22	21.36
	N2	5	4.85
	N3	3	2.91
M	M0	95	97.94
	M1	2	2.06

Note: There were a total of 103 TNBC patients, and the median follow-up was 5.3 years. The median age was 52 (range: 26, 80). Among them, 22 had metastasis, 3 had recurrence, and 24 died during the follow-up.

Table S3. Summary of metastasis-free survival between marker groups in triple negative breast cancer group

Variable	level	N	Events	P
age	<=52.3	52	12	0.591
	> 52.3	51	10	
DEAR1_Cytoplasm	<=43	53	16	0.042
	> 43	50	6	
DEAR1_Membrane	<=26	52	14	0.265
	> 26	51	8	
SNAI2_Cytoplasm	<=4	50	11	0.997
	> 4	49	10	
SNAI2_Nuclear	<=0	87	15	0.025
	> 0	12	6	
